# Fundamentals of lipoprotein(a) request and quantification in the clinical laboratory

**DOI:** 10.1515/almed-2025-0034

**Published:** 2025-03-03

**Authors:** Teresa Arrobas Velilla, Carla Fernández Prendes, Núria Amigó Grau, Pilar Calmarza, Silvia Camós Anguila, Beatriz Candas Estébanez, María José Castro Castro, David Ceacero, Irene González Martínez, María Martín Palencia, José Puzo Foncillas, Carlos Romero Román, on behalf of the SEQCML Working Group on Lipoproteins and Cardiovascular Diseases

**Affiliations:** Virgen Macarena University Hospital, Seville, Spain; Analysis and Clinical Biochemistry Service, Laboratori Clínic Metropolitana Nord, Hospital Universitari Germans Trias i Pujol, Badalona, Barcelona, Spain; Department of Basic Medical Sciences, Rovira i Virgili University, Reus, Spain; Center for Biomedical Research in Diabetes and Associated Metabolic Diseases (CIBERDEM), Instituto de Salud Carlos III, Madrid, Comunidad de Madrid, Spain; Biosfer Teslab, Reus, Spain; Service of Clinical Biochemistry, Miguel Servet University Hospital, Zaragoza, Aragón, Spain; Centre for Networked Research in Cardiovascular Diseases (CIBERCV), Instituto de Investigacion Sanitaria Aragon, Zaragoza, Spain; Service of Clinical Biochemistry- Laboratori Clínic Girona, Hospital Universitari de Girona Doctor Josep Trueta, Catalunya, Spain; Clinical Laboratory, Hospital of Barcelona, Barcelona, Catalunya, Spain; Faculty of Medicine, UVic-UCC, Vic, Spain; Faculty of Sciences, UVic-UCC, Vic, Spain; Biochemistry Core, Laboratori Clínic Territorial Metropolitana Sud, Bellvitge University Hospital, L’Hospitalet de Llobregat, Spain; Service of Clinical Biochemistry, 12 de Octubre University Hospital, Madrid, Autonomous Community of Madrid, Spain; Service of Clinical Biochemistry, University Hospital of Burgos, Burgos, Castilla y León, Spain; Service of Clinical Biochemistry, Unit of Lipids, Hospital General Universitario San Jorge de Huesca, General University Hospital, Huesca, Spain; Faculty of Life Sciences and Sports, Huesca, Spain; General University Hospital of Albacete, Albacete, Castilla-La Mancha, Spain

**Keywords:** cholesterol, determination, isoforms, lipoprotein(a), recommendations

## Abstract

Cardiovascular diseases keep being the leading cause of mortality in Spain. Efforts should be intensified to identify new risk factors that may contribute to increasing cardiovascular risk. Lipoprotein(a) (Lp(a)) has been associated with a higher risk for developing aortic valve stenosis, heart failure, ischemic stroke, ischemic heart disease and peripheral arterial disease. Hyperlipoproteinemia(a) is a common health problem. Between 10 and 30 % of the world population have Lp(a) values exceeding 50 mg/dL. The scientific evidence provided in the recent years confirms an independent association between Lp(a) and the risk for having an arteriosclerotic cardiovascular event. This finding, added to the emergence of new specific therapies for reducing Lp(a) has raised interest in the quantification of this lipoprotein. The objective of this paper was to perform a review of the evidence available to identify the patients who will benefit from undergoing Lp(a) testing and determine the recommended quantification methods, the desirable concentrations, and the role of Lp(a) determination in reclassifying the cardiovascular risk of patients.

## Introduction

Cardiovascular and circulatory system diseases keep being the leading cause of mortality in Spain [1]. In addition, a significant proportion of the population develops early or recurrent cardiovascular events despite regular follow-up of classic cardiovascular risk factors (RFs) such as arterial hypertension, diabetes mellitus (DM) or smoking, the availability of optimal drug therapies for reducing low density lipoprotein cholesterol (LDL-C) and close monitoring [2], 3].

This suggests the presence of other non-classic or independent RFs that may either contribute to increasing cardiovascular risk (CVR) or, in patients with adequate control of classic RFs, confer a residual risk for the development of an atherosclerotic cardiovascular event [3].

Lipoprotein(a) (Lp(a)) plays a major role in the development of the atherosclerotic plaque. Elevated lipoprotein(a) (Lp(a)) concentrations have been associated with a higher risk of developing aortic valve stenosis, heart failure, ischemic stroke, ischemic heart disease and peripheral arterial disease [2]. Lp(a) has proinflammatory, proatherosclerotic and procalcific activity that may be partly related to oxidized phospholipids (OxPL), preferentially transported by Lp(a) in plasma [4].

Hyperlipoproteinemia(a) is a common health problem. It is estimated that 10–30 % of the world population has Lp(a) concentrations >50 mg/dL, of which 148 million live in Europe [5]. The Spanish Familial Hypercholesterolemia Cohort Study (SAFEHEART) reveals that around 30 % of patients with familial hypercholesterolemia (FH) had Lp(a)>50 mg/dL (125 nmol/L) [6]. This finding is consistent with the results reported for Extremadura and Andalusia, with 29.58 % of patients exhibiting Lp(a) concentrations >50 mg/dL (125 nmol/L) and 1.52 % showing >180 mg/dL (430 nmol/L) [7].

Lp(a) was first described by the Norwegian physician Kåre Berg in 1963 during a study where rabbits were immunized using low-density lipoproteins. Lp(a) was considered an antigenic variant of LDL-C [8]. It was not until 1970 that a new electrophoretic lipoprotein fraction was identified, which was initially-called pre-β-lipoprotein after its migration positioning in agarose gel, and later named Lp(a) [9], 10]. Since then, evidence has been provided of the role of Lp(a) as a genetic RF in coronary diseases [11]. Lp(a) is currently considered a major hereditary RF in cardiovascular disease [12].

**Figure 1:**
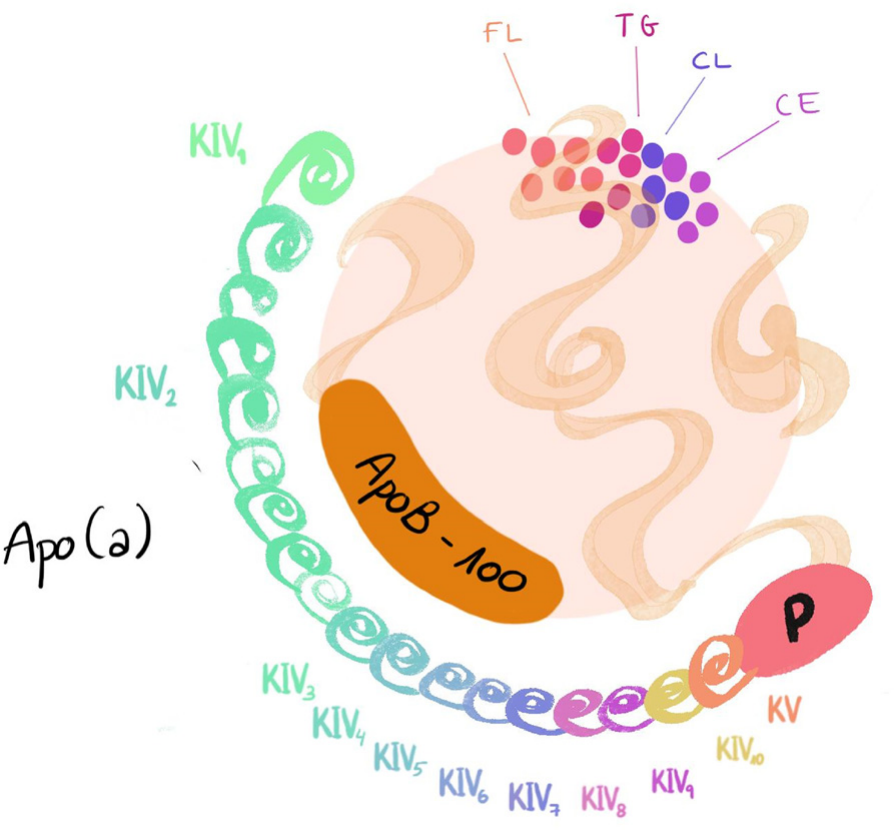
Structure of a lipoprotein(a) molecule.

Lp(a) is a plasma lipoprotein with a LDL-like structure in terms of size, lipid composition and presence of apolipoprotein B100 (apo B100). Additionally, Lp(a) contains a highly glycosilated polymorphic protein called apolipoprotein (a) (apo(a)) bound covalently to apo B10 in an equimolar proportion of 1:1 [10]. It is synthetized and secreted by the liver (Figure 1) [13], 14].

The gene encoding Lp(a) is located in positions 26 and 27 of the long arm of chromosome 6 (6q26–27). It is characterized by a high rate of polymorphism and consists of a variable number of exons and a protein domain known as “kringle” (k) [15], which evolved from a plasminogenic gene (PLG). The plasminogen has a structure of five loops called kringles and a protease domain. Unlike plasminogen, apo(a) lacks KI, KII and KIII, only has a copy of KV, an inactive serine-protease like domain, and ten subtypes of KIV (from KIV1 to KIV10) [16]. The KIV2 subtype is predominant as it repeats in multiple copies, which explain heterogeneity in the size of apo(a) isoforms and is the main challenge to apo(a) quantification [17].

The molecular weight of apo(a) ranges from 275 to 800 kDa, which is due to the presence of over 40 different isoforms. This variability is due to the fact that apo(a) isoforms are determined by the number of KIV2 repeats in the gene encoding this protein, ranging from 3 to over 40 repeats [13]. Two alleles very rarely have exactly the same number of copies in their genomes. Up to 80 % of patients have two different apo(a) isoforms [17].

The high biological between-subject variability of Lp(a) concentrations is due to the copy number variant (CNV) in the locus of the LPA gene. This explains 70–90 % of differences in concentrations between individuals [18], 19].

Additionally, Lp(a) concentration is inversely related to the length of the apo(a) isoform. Hence, the higher the number of copies of KIV2, the longer will be the length of the apo(a) isoform and the lower will be plasma Lp(a) concentration [19]. The high concentrations of the shortest Lp(a) isoform, with very few KIV2 copies strongly correlates with a higher cardiovascular risk [20].

It is widely accepted that Lp(a) concentration is determined by its production and secretion rather than its catabolism. However, the site of assembly and clearance has not yet been confirmed [21]. The apo(a) genotype determines both, the apo(a) synthesis rate and its length, accounting for 90 % of plasma concentration. Although the assembly site of this lipoprotein is still unknown, its components are mostly synthetized in hepatocytes [17], 21]. Lp(a) clearance pathways are not well understood. Some authors suggest a two-step clearance mechanism by which Lp(a) releases apo(a) into the circulation, although the reactions involved in this process are unknown. Other authors suggest that Lp(a) may be residually cleared through LDL and scavenger receptors [18], 21].

Other genetic factors that may affect Lp(a) concentrations include single-nucleotide polymorphisms (SNP), being rs3798220 and rs10455872 the most extensively studied, along with pentanucleotide repeat polymorphisms [15]. Lp(a) levels significantly vary across races and ethnic groups, with black individuals having significantly higher levels. This variability is more significant that the one observed in other cardiovascular biomarkers [22].

Environmental factors play a minor role in variations in plasma Lp(a) concentrations. However, some secondary factors might influence concentrations [23]. Patients with chronic kidney disease (CKD), proteinuria within nephrotic range and clear hypothyroidism often have elevated levels of Lp(a). In contrast, patients with liver disease exhibit reduced concentrations [24]. Whereas men have stable Lp(A) concentrations throughout life [7], postmenopausal women develop increased levels of Lp(a) as compared to their premenopausal status. Levels may return to normal with hormone replacement therapy [19], 23].

## Who should undergo Lp(a) testing?

Plasma Lp(a) concentrations remain relatively stable throughout life due to their genetic predetermination and are hardly influenced by lifestyle. Concentrations can be 5–10 % higher in women than in men [19].

Most clinical guidelines do not recommend screening the general population [24], [25], [26], [27], [28], [29]. This occurs because despite the strong association between elevated Lp(a) concentrations and ASCVD and AVD, there is no solid scientific evidence available demonstrating a direct relationship between reducing Lp(a) and a decrease in cardiovascular events, regardless of LDL-C concentration. This is also due to the lack of standardized measurement methods. Table 1 displays recommendations for Lp(a) screening according to the different scientific societies [25], [26], [27], [28], [29], [30], [31], [32], [33].

**Table 1:** Recommendations for Lp(a) screening according to the different scientific societies.

Clinical practice guidelines	Applicable screening recommendations
2019 ACC/AHA (27)	– Family history of premature ASCVD not explained by major RFs.
2021 NLA (28)	In adults, Lp(a) testing is recommended as initial evaluation in the following cases: – Personal history of premature ASCVD – Family history of premature ASCVD or elevated Lp(a) – LDL-C>190 mg/dL – Suspicion of FH – Very high risk for ASCVD – Patients on lipid-lowering treatment at maximum estatin dose ± ezetimiba, with LDL-C levels >70 mg/dL that could benefit from PCSK9 inhibitors – In young adults (<20 years): – Upon clinical suspicion or genetically-confirmed FH – Subjects with first-degree relatives with cardiovascular disease (CVD) – Ischemic stroke of unknown etiology – First-degree relatives with elevated Lp(a)
2019 HEART UK (25)	– Personal or family history of premature ASCVD (<60 years) – First-degree relative with elevated levels of Lp(a) (>200 nmol/L) – Familial hypercholesterolemia (FH) or another genetic dyslipidemia – Calcified aortic valve stenosis – 10-year medium/limit risk of ASCVD, for risk reclassification
2021 NSFA (29)	Lp(a) testing is recommended in the following cases: – Patient with a high CVR or a family history of premature coronary heart disease – FH diagnosis – Suspicion of atherogenic dyslipidemia, diabetes type 1, diabetes type 2 or CKD
	Lp(a) testing is not recommended in the presence of liver failure, inflammation or concurrent disease. Testing should not be repeated if it was performed in adequate baseline conditions.
2019 ESC/EAS (30)	– Lp(a) testing should be considered at least once in a lifetime in adults to identify subjects with an inherited Lp(a) concentration >180 mg/dL (>430 nmol/L) with a whole-life risk fo ASCVD, equivalent ot the risk of heterozygous familial hypercholesterolemia (FHe). – Lp(a) testing should be considered in selected patients with a family history of premature CVD and for reclassification of patients in the moderate-to-high risk limit.
2021 Canadian Cardiovascular Disease (31)	– Test Lp(a) at least once in life – Subjects within the Framingham medium risk category (10–19 %) – Family history of premature CVD
2023 Consensus statement for determination and reporting of lipid profile in Spanish clinical laboratories (32)	Test Lp(a) at least once in life
	This test is especially relevant in patients with premature CVD, familial hypercholesterolemia, poor response to statins, aortic stenosis or recurrent ischemic events, and in relatives of patients with elevated Lp(a).
2022 European Society of Arteriosclerosis (26)	Lp(a) should be measured at least once in life in adults, preferably in the first lipid profile test, to identify subjects with a high cardiovascular risk. Testing would facilitate the identification of adults with very high inherited levels of Lp(a)>180 mg/dL (>430 nmol/L) with a potential high risk of ASCVD equivalent to the risk of heterozygous familial hypercholesterolemia (FHe).
2024 Consensus on Lipoprotein(a) of the Spanish Society of Arteriosclerosis (33)	First lipoprotein(a) testing is recommended in the following cases: – Patients with a clinical manifestation of (patients on secondary prevention) – AVS of any territory, especially in early onset disease – Aortic stenosis (calcified) in subjects <65 years – Familial hypercholesterolemia (confirmed or clinical suspicion) regardless of genetic screening result for FH – First-degree relatives with elevated Lp(a) Inevitably, if the index case has a Lp(a)>200 nmol/L, or the index case has >100 nmol/L and other CV risk factors – Familial history of early CVS of unkwnon etiology (first-degree relatives) – Poor response to statin treatment <20 % reduction of c-LDL with medium or high dose statin therapy – First evaluation of CV risk, to improve risk stratification – As a general recommendation, testing Lp(a) at least once in life coinciding with a blood draw for lipid profile quantification is advisable for all the population

ACC/AHA, American College of Cardiology/American Heart Association; NLA, National Lipid Association; HEART UK, HEART UK, The Cholesterol Charity; NSFA, New French Atherosclerosis Society; ESC/EAS, European Society of Cardiology/European Atherosclerosis Society.

Guidelines and consensus statements generally suggest measuring Lp(a) at least once in life. This recommendation is based on the consideration that elevated Lp(a) concentrations are a RF. Moreover, guidelines agree that Lp(a) should be measured in the presence of a family history of premature CVD [15], [24], [25], [26], [27], [28], [29], [30], [31].

The inclusion of Lp(a) when assessing global cardiovascular risk can also improve risk classification. Results should be interpreted considering other RF to avoid underestimating the absolute overall risk for a cardiovascular event. This approach may help identify individuals with slightly elevated Lp(a) levels that may have a higher risk for ASCVD that is not appropriately indicated by the SCORE system or other lipid or lipoprotein tests. Therefore, Lp(a) testing should be considered in patients whose estimated 10-year risk for ASCVD is close to the moderate-high risk cut-off [34].

## Should Lp(a) be measured in children and adolescents?

The genetic variations that determine high plasma Lp(a) concentrations are present from birth. Several studies demonstrate that Lp(a) is low at birth and reaches adult levels within the first two years of life [30]. Early detection of elevated Lp(a) concentrations can be clinically relevant and support the recommendation to have healthy life habits to minimize the development of atherogenesis, which starts in childhood [34], 35].

Recommendations for children and adolescents are limited and only support selective Lp(a) testing by family cascade screening [36], 37]. Other guidelines recommend measuring Lp(a) several times, as levels can increase in adulthood [38]. The Expert Panel on Integrated Guidelines for Cardiovascular Health and Risk Reduction in Children and Adolescents [39] recommends measuring Lp(a) in children ≥2 years with a family history of ischemic/hemorrhagic cerebrovascular accident and a family history of CVD not explained by the classic FRs.

The European Society of Cardiology consensus statement [30] recommends Lp(a) screening in young adults in the presence of a family history of ischemic cerebrovascular accident or premature ASCVD or elevated Lp(a) without other identifiable RFs.

The NLA recommends [28] selective Lp(a) screening in young subjects <20 years with FH, since they are at a high risk for accelerated ASCVD. This risk increases in the presence of elevated LDL-C and Lp(a), a family history of premature CVD in first-degree relatives, ischemic stroke of unknown etiology, and elevated Lp(a) concentrations in parents or siblings. Some authors recommend cascade screening in subjects with a family history of very elevated Lp(a) or a personal or family history of ASCVD and FH [19]. The NLA recommends reverse cascade screening in the presence of elevated levels of Lp(a) in children [28].

A few studies suggest an association between Lp(a) and the incidence of arterial ischemic accident and venous thromboembolism/venous thrombosis in children, with risk doubling with levels of Lp(a)>30 mg/dL [26].

## When should measurement be repeated?

Plasma Lp(a) concentrations are primarily determined by genetic factors and remain stable throughout life. However, only a few longitudinal studies are available assessing potential variations in Lp(a) concentrations over time or establishing the optimal timing for determination.

Two recent studies revealed changes in patient risk categorization after considering their Lp(a) values and other factors such as sex. In these studies, men exhibited a slightly higher variability than women. Menopause [40] and dependence on basal concentration [41] were identified as conditioning factors of Lp(a) variability.

Hence, the suitability of measuring Lp(a) once in a lifetime to assess cardiovascular risk in all adults is still unclear. Repeat Lp(a) measurement should be considered especially in patients with Lp(a) concentrations in “the grey zone” [41]. Likewise, a repeat measurement should be indicated upon suspicion of changes in Lp(a) concentrations due to secondary causes. These causes include acute processes with associated inflammation, chronic kidney disease (CKD), nephrotic syndrome, chronic liver disease, hypothyroidism, diabetes mellitus (DM) and postmenopausal status. Repeat measurements can also be useful for monitoring response to therapeutic interventions aimed at reducing plasma Lp(a) concentrations.

Table 2 shows non-genetic factors that may influence Lp(a) concentrations [42].

**Table 2:** Non-genetic factors that may influence Lp(a) concentration.

Interventions and conditions		Association with Lp(a) levels
1	**Diet**	
	a. Replacement of dietary saturated fat with carbohydrates or unsaturated fat	∼8–20 % increase
	b. Low carbohydrate and high saturated fat diet	∼15 % decrease
	c. Alcohol consumption	No association, negligible decrease
	d. Fasting	No association at all
2	**Physical activity and exercise**	No association or minimal association
3	**Sex, hormones** and **association conditions**	
	a. Sex	No association or higher levels in women
	b. Sexual hormones (endogenous)	No association or minor association
	c. Postmenopausal hormone replacement therapy	∼20–25 % decrease
	d. Hyperthyroidism	Lp(a) decrease; hyperthyroidism treatment increases Lp(a) by 20–25 %
	e. Hypothyroidism.	Elevated Lp(a); hypothyroidism treatment reduces Lp(a) by 5–20 %.
	f. Growth hormone replacement therapy	∼25–100 % increase
	g. Pregnancy	Increase (up to 100 %)
	h. Menopause	Increase
	i. Growth hormones	Increase (up to 100 %)
4	**Chronic kidney disease**	
	a. Chronic kidney disease and hemodialysis	Elevated Lp(a); 2–4 times higher in carriers of large isoforms
	b. Continuous ambulatory peritoneal dialysis	∼twice as high as in controls
	c. Nephrotic syndrome	∼3–5 times as high as in controls
	d. Kidney transplant	Significant reduction; return almost to normal
5	**Liver disease**	
	a. Hepatocellular injury	Decreases as disease progresses
	b. Non-alcoholic fatty liver disease	Inconsistent association across population groups
6	**Inflammation**	
	Inflammation	Increase

## What is the cut-off for elevated Lp(a)?

Determination of Lp(a) concentrations by a standardized assay is the gold standard method for estimating atherogenic risk associated with this lipoprotein. The use of SNP to estimate risk does not provide any added value to the measurement of this biologically active protein [25], 26].

A consensus cut-off for universal risk [40] has not been established due to the heterogeneity of the measurement methods and units used. Some authors highlight the need for establishing specific ranges as a function of ethnicity and comorbidities (liver diseases, CKD, DM) has also been highlighted. Table 3 displays the cut-off point recommended by different scientific societies [25], [26], [27], [28], [29], [30], [31], [32].

**Table 3:** Cut-offs indicating cardiovascular risk due to Lp(a) elevation recommended by different scientific societies.

Clinical guidelines	Upper limits of normality for cardiovascular risk due to Lp(a) elevation
2018 ACC/AHA	>125 nmol/L (50 mg/dL)
2019 NLA	>100 nmol/L (40 mg/dL)
2019 ESC/EAS	>430 nmol/L (>180 mg/dL) risk threshold equivalent to HFHe
2019 HEART UK	Minor risk: 32–90 nmol/L
	Moderate risk: 90–200 nmol/L
	High risk: 200–400 nmol/L
	Very high risk: >400 nmol/L
2021 Canadian Cardiovascular Disease	>125 nmol/L (50 mg/dL)

ACC/AHA, American College of Cardiology/American Heart Association; NLA, National Lipid Association; ESC/EAS, European Society of Cardiology/European Atherosclerosis Society; HEART UK, HEART UK, The Cholesterol Charity.

## Measurement methods and their limitations

Different methods are currently available for the quantification of Lp(a) concentrations, including immunoassays, electrophoresis and mass spectrophotometry. However, these methods have some limitations due to the heterogeneity of Lp(a) isoforms, which may vary in size and structure [13], 15], 17]. This variability may lead to differences in the results obtained by different methods, which hinders comparison of data across studies.

The most extensively used methods are immunoturbidimetric and nephelometric immunoassays [43], 44]. For an analytical method to be adequate, the following factors should be considered:

– Number of variable KIV2 repeats in the apo(a) molecule.
– Use of polyclonal antibodies that recognize different apo(a) epitotes, including repeat sequences in variable number. This may lead to overestimate or underestimate Lp(a) concentration as a function of the presence of small or large Lp(a) isoforms.

Measuring levels of Lp(a) accurately is challenging due to the high variability of apo(a) sizes resulting from the variable number of KIV2 repeats. Selecting assay calibrators with the same apo(a) size present in the individual samples to be analyzed is virtually impossible. This may lead to an overestimation of Lp(a) values in samples with larger Lp(a) molecules than those in the calibrator, and to an underestimation of Lp(a) values in samples with smaller Lp(a) molecules than those in the calibrator [14], 43].

The mass of the measured particles will not reflect the number of Lp(a) particles due to heterogeneity in size. The calibrators of turbidimetric and immunonephelometric assays are generally selected for their high Lp(a) concentrations. However, the isoforms present in calibrators may be primarily composed of small apo(a) isoforms. This results in an overestimation of Lp(a) values in most samples. Additionally, the total mass of the heterogeneous Lp(a) particle cannot be calculated accurately, since it involves quantifying all Lp(a) components separately, including the protein, multiple lipids and carbohydrates [43], 45].

The methods available for measuring molar Lp(a) concentration determine apo(a), the particle of the main component that identifies the Lp(a) particle, without the bias caused by the size of the particle. In contrast, mass assays measure variable levels of all Lp(a) mass components, thereby adding sensitivity to differences in size across apo(a) isoforms [43].

Moreover, the proportion of these Lp(a) components may differ across patients, which adds inaccuracy to measurements. Therefore, mass assays, which provide results in mg/dL, have an inherent limitation in accurately measuring the concentration of circulating Lp(a) particles and may not have sufficient analytical quality from a clinical point of view [43].

To minimize limitations related to the measurement of Lp(a) concentration, it is important to follow the criteria and recommendations of the International Federation of Clinical Chemistry and Laboratory Medicine (IFCC) and the World Health Organization (WHO) to ensure accurate, reliable results [46].

The IFCC Working group for Apolipoproteins by Mass Spectrometry (IFCC WG APO-MS) is developing new measurement methods for a more accurate molecular characterization of apo(a). This process involves the development of primary and secondary reference standards for apolipoproteins, including Lp(a). For such purpose, a reference procedure of measurement based on liquid chromatography-mass spectrometry (LC-MS/MS) is used. This method will make it possible to reliably define apo(a) composition. Additionally, it will guarantee that the commercially-available measurement methods for Lp(a) or apo(a) are truly traceable to the International System [47].

The characterization of a secondary reference material for use by the manufacturers of commercially-available methods will help assign an accuracy-based target value of Lp(a) to its calibrators. In view that determination of total Lp(a) mass is very heterogeneous and challenging, a target value in nmol/L was assigned to Lp(a) based on the size of the apo(a) polymorhism, which is the Lp(a) component most commonly measured directly by immunoassays [48].

In the study for the standardization of reference materials for measuring Lp(a) concentration conducted by Dikaios et al. [49], the authors assessed the correlation between the candidate gold standard method and immunoassay-based measurement procedures and the commutability of the different reference materials. The results show the need for standard operating procedures for the measurement of Lp(a) concentration to improve patient care.

In some studies, the method developed by Northwest Lipid Metabolism and Diabetes Research Laboratories (NLMDRL) (University of Washington) was used as the gold standard for measuring Lp(a) concentration [50], 51].

Determination of Lp(a) concentration by a standardized assay is the method of choice to estimate the risk associated with this atherogenic lipoprotein. The assays that display Lp(a) concentration in mg/dL indicate the mass of Lp(a) particles of different sizes. Conversely, the assays that express it in nmol/L reflect the actual number of particles. In the light that the most appropriate unit of measurement of Lp(a) is concentration in nmol/L, converting results from mg/dL into nmol/L is not recommended, as all conversion factors directly depend on the Lp(a) isoform.

The following steps should be taken for an improved standardization of Lp(a) measurement:

– Verify that the assay accuracy is certified by the Northwest Lipid Metabolism and Diabetes Research Laboratories, Seattle.
– Do not convert results from nmol/L into mg/dL.
– Use external quality assurance programs that distribute samples with a known apo(a) isoform composition. Ensure that Lp(a) values have been assigned by a validated method that does not rely on the apo(a) size polymorphism and that calibration is traceable to the WHO/IFCC reference material.
– External quality control samples will fall within the range of clinically relevant concentrations, 90–200 nmol/L.

At present, advanced lipoprotein markers such as the number of particles containing apoB100 have gained relevance, especially when assessing residual cardiovascular risk. Nuclear magnetic resonance (NMR) is the most effective technique for determining particle count or concentration. However, NMR does not separate Lp(a) concentration from that of LDL [52], [53], [54]

Considering the aforesaid, the comparability of the different analytical techniques is limited. A recent review of assays with Five-Point calibration revealed significant variations between laboratories and between assays. Differences are only partly explained by apo(a) size polymorphism [55].

The most effective quantification technique would be to obtain an antibody against a single non-repeating epitope in apo(a) that recognizes every Lp(a) particle once and presents results in nmol/L. Marcovina et al. [56] developed an immunoassay that uses a monoclonal antibody targeted against a single antigenic site, present in KIP subtype 9.

**Figure 2:**
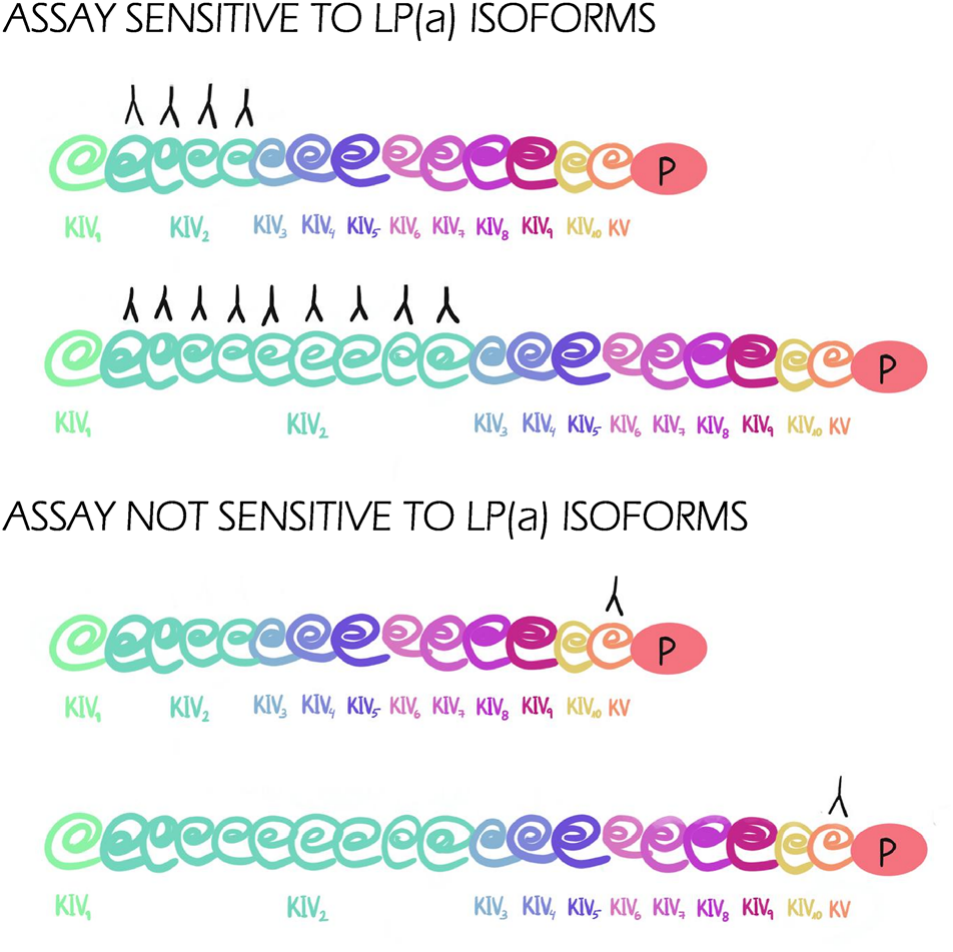
Diagram of methodological differences across assays sensitive and not-sensitive to different Lp(a) isoforms.

Figure 2 displays the use of an analytical method that is sensitive to the different Lp(a) isoforms as compared to a non-sensitive method.

## Is correction for Lp(a) recommended for LDL-C determination?

One of the main components of the Lp(a) particle is cholesterol, which accounts for 30–45 % of particle components. In patients with very elevated levels of Lp(a), cholesterol may influence LDL-C estimate by the traditional formula. Hence, LDL-C corrected for Lp(a) is occasionally used. The Friedewald formula modified by Dahlen [57], with Lp(a) values quantified in mg/dL, has been used in some studies such as the FOURIER study [58]. This formula requires Lp(a) to be measured in mg/dL or be converted from mmol/L into mg/dL.

In current practice, the methods used for LDL-C quantification measure both, cholesterol contained in LDL and in Lp(a). An advanced, sensitive, rapid method is available that provides information on the Lp(a) mass/cholesterol relationship. This technique allows for a more accurate estimation of LDL-C and a reevaluation of its role in clinical medicine. The LDL-C is determined following isolation of the Lp(a) on magenitc beads linked to monoclonal antibody LPA4 recognizing apolipoprotein(a). This assay does not detect cholesterol in plasma samples lacking Lp(a) and is linear up to Lp(a) concentrations of 747 nM.

It should be taken into account that subjects with elevated levels of Lp(a) usually exhibit a poor response to lipid-lowering drugs such as statins. Indeed, poor response to lipid-lowering therapies should raise suspicion of elevated Lp(a) concentration and testing should be performed [59].

## What is the role of the laboratory on the analytical report?

An important detail of the EAS (European Atherosclerosis Society) document [26] is the relevance conferred to the role of the clinical laboratory in analytical and post-analytical processing. Hence, the EAS suggest including the following details in laboratory reports:

– Type of technique used for a correct interpretation of discrepant results throughout follow-up.
– Integrate alerts or interpretative comments about the role of Lp(a) in ASCVD, suggest requesting family cascade screening and consider referral to a Lipid Control Unit.
– Consider secondary causes that may have caused Lp(a) elevation.

Find below some examples of interpretative comments for Lp(a):

– Lp(a)=120–180 mg/dL. Very high risk for myocardial infarction and aortic valve stenosis [60], 61].
– Lp(a)>180 mg/dL. Confers a higher risk for LP equivalent to the risk associated with HFHe [60], 61].
– Lp(a) confers a higher risk for ASCVD and does not change with standard lipid-lowering therapies. Lp(a) monitoring is not recommended, except in therapies targeting PCSK9 [60], 61].
